# Calendar

**DOI:** 10.1155/1995/28098

**Published:** 1995


					Calendar
Date Venue Event Contact Address
1995 Ulm,
June Germany
12-14
June Ljubljana,
19 Slovenia
June Ljubljana,
19-23 Slovenia
August Moscow,
7-11 Russia
September
7-9
September
13-16
September
17-21
October
11-14
Pilsen,
Czech
Republic
Pavia,
Italy
Berlin,
Germany
Tenerife,
Canary
Islands,
Spain
VIIIth Postgraduate Course
of Pancreatic Surgery,
Surgery of the Pancreas
Standards and New Procedures
Prof. H. G. Berger, Congress Secretariat,
University of Ulm, Steinhoevelstrasse 9, Ulm
D-89075, Germany.
Phone: +731 502-72 16 Fax: + 731 502 72 09
Hepatobiliary School,
3rd Postgraduate
Course on Hepatology
Saga Markovic, Institute of Oncology, Zalogka 2
Ljubljana, 61000 Slovenia.
Phone: + 386 61 30 28 28 Fax: + 386 61 30 28 28
Hepatobiliary School,
3rd Postgraduate Course
on Hepatobiliary Surgery
Eldar Gadzijev, Clinical Centre, Dept. of
Gastroenterologic Surgery, Zalogka 7
Ljubljana, 61000, Slovenia.
Phone: + 386 61 32 22 82 Fax: + 386 61 31 60 96
The First International Organizing Secretariat CIS, 1-aya
Congress of Surgeons in Brestskaya St., 17, Moscow 12540RF, Russia.
Moscow, Devoted to Peritonitis Phone: +07095 251-0329 Fax: +07095 251-3789
and Hepato- Biliary-Pancreatic
Surgery
1st Czech Surgical Congress Congress Secretariat, University Hospital,
with International Participation Department of Surgery, Dr. E. Bense 13, Pilsen
Acute Non-Traumatic 405 99, Czech Republic.
Emergencies in the areas Phone: +42 19 273336 Fax: +42 19 27333
of Neck, Abdomen
2nd Alps Adria Congress
on Hepato- Pancreato-Biliary
Surgery
Rosangela Quita, Organizing Secretariat,
MGR Congressi, Via Servio Tullio 4, Milan
20123, Italy.
Phone: +02 430071 Fax: +02 48008471
4th United European
Gastroenterology Week
R. Arnold, C/o Department of Internal Medicine,
Phillips University, P.O. Box 23 60, Marburg,
D-25033, Germany.
Phone: +49 6421 28 2319 Fax: +496421 28 23 19
XIX European Federation
Congress of the International
of the College of Congress
Spanish Section of the I.C.S.
Prof. D. Antonio Alarc6-Hernfi Departmento de
Cirugifi, Hospital Universitario de Canarias,
Carretera. General la Cuesta-Taco S/N, La
Laguna, Tenerife 38320, Canary Islands, Spain.
Phone: + 34 9 22-603436 Fax: + 34 9 22 603445
213
214 CALENDAR
Date Venue Event Contact Address
December
5-7
1996
March
20-22
Hong Kong 10th International Workshop
on Therapeutic Endoscopy
10th International Meeting on
Therapeutic Endoscopy
Brighton,
UK
British Society for
Gastroenterology, Pancreatic
Section International
Symposium
Sydney Chung, Endoscopy Centre, Prince of
Wales Hospital, The Chinese University of
Hong Kong, Shatin N.T., Hong Kong.
Phone: + 852 2632 2233 Fax: + 852 2632 0075
Mr. Colin Johnson, University Surgical Unit
Tremona Road, Southampton, S09 4XY, UK.

				

